# Empagliflozin in Patients With Heart Failure: A Systematic Review and Meta-Analysis of Randomized Controlled Trials

**DOI:** 10.3389/fcvm.2021.683281

**Published:** 2021-06-22

**Authors:** Deng Pan, Lin Xu, Pengfei Chen, Huiping Jiang, Dazhuo Shi, Ming Guo

**Affiliations:** ^1^Graduate School of Beijing University of Traditional Chinese Medicine, Beijing, China; ^2^National Clinical Research Center for Chinese Medicine Cardiology, Xiyuan Hospital, China Academy of Chinese Medical Sciences, Beijing, China; ^3^Gynecological Department of Traditional Chinese Medicine, China-Japan Friendship Hospital, Beijing, China; ^4^Graduate School of China Academy of Chinese Medical Sciences, Beijing, China; ^5^No.3 People's Hospital of Heze, Shandong, China

**Keywords:** empagliflozin, sodium-glucose cotransporter 2 inhibitors, heart failure, systematic review, cardiovascular

## Abstract

**Purpose:** The purpose of the study is to evaluate the effect of empagliflozin in patients with heart failure (HF).

**Method:** We performed a systematic search of PubMed, EMBASE, and the Cochrane Library database through January 20, 2021. Randomized controlled trials (RCTs) were included that compared empagliflozin and placebo in patients with HF. Dichotomous variables were expressed as risk ratios (RRs) with 95% confidence intervals (CIs). Continuous variables were calculated and expressed as mean differences (MD) and standard deviation (SD). Meta-analysis was conducted using a random-effects model on outcomes with high heterogeneity.

**Results:** Seven studies were included in our meta-analysis (*n* = 5,150). Significant differences were observed in a composite of cardiovascular death or hospitalization for worsening heart failure [RR: 0.77 (95% CI 0.68–0.87); *I*^2^ = 18%; *P* < 0.0001), hospitalization for worsening heart failure [RR: 0.71 (95% CI 0.61–0.82); *I*^2^ = 0%; *P* < 0.00001], changes in Kansas City Cardiomyopathy Questionnaire (KCCQ) score [MD: 1.70 (95% CI 1.67–1.73); *I*^2^ = 0%; *P* < 0.00001], and changes in body weight [MD: −1.43 (95% CI −2.15 to −0.72); *I*^2^ = 84%; *P* < 0.0001) from baseline. However, empagliflozin did not show a better change in the 6-min walk test (6MWT) [MD: 34.06 (95% CI −29.75–97.88); *I*^2^ = 97%; *P* = 0.30] or NT-proBNP [MD: −98.36 (95% CI, −225.83–29.11); *I*^2^ = 68%; *P* = 0.13] from baseline.

**Conclusion:** The findings suggest that empagliflozin was effective in reducing a composite of cardiovascular death or hospitalization for worsening heart failure. Further well-designed RCTs are needed to evaluate the long-term effect of empagliflozin in patients with HF.

**PROSPERO:** CRD42021231712.

## Introduction

Heart failure (HF) leads to a high economic burden, and the prevalence of HF is also increasing gradually (1, 2). It has been estimated that over 26 million people around the world are suffering from heart failure (3). According to the guidelines (4) for HF patients, it is recommended to apply angiotensin-converting enzyme inhibitors, beta-blockers, mineralocorticoids, or aldosterone receptor antagonists. Currently, new drugs such as sodium–glucose cotransporter 2 inhibitors (SGLT2i) and vericiguat also show positive effects across the spectrum of HF (5).

SGLT2i has been used to treat type 2 diabetes mellitus (6, 7). In recent years, SGLT2i has shown a better effect than placebo on the outcomes of all-cause mortality and heart failure hospitalization in patients with type 2 diabetes mellitus in clinical trials, regardless of the presence or absence of heart failure (8, 9). In patients with diabetes mellitus, SGLT2i reduced the risk of all-cause mortality and heart failure hospitalizations by 23% (10). Empagliflozin, one of the frequently used SGLT2i, has been proven to be effective in reducing HF hospitalizations, cardiovascular deaths, and biomarkers in patients with HF (11). However, the results in related articles showed heterogeneity in some outcomes, for example, N-terminal pro-brain natriuretic peptide (NT-proBNP) (12, 13) and the 6-min walk test (6MWT) (14, 15). The effect of empagliflozin in patients with HF has not been evaluated specifically. Previous studies mostly concentrated on SGLT2i rather than empagliflozin. According to previous large-sample trials, empagliflozin also showed different results of a composite cardiovascular endpoint (cardiovascular deaths, non-fatal myocardial infarction, or non-fatal stroke) when compared with dapagliflozin, which means that different drugs may present different results, although all of them belong to SGLT2i (9, 16). Thus, we aim to perform a systematic review and meta-analysis of randomized controlled trials (RCTs) to evaluate the effect of empagliflozin on patients with HF.

## Methods

Our systematic review and meta-analysis were conducted based on preferred Reporting Items for Systematic Reviews and Meta-Analyses (PRISMA) guidelines. Ethical approval was not required. The study protocol was registered in the PROSPERO international prospective register of systematic review (CRD42021231712).

### Search Strategy

Our systematic review and meta-analysis were conducted according to the PRISMA guidelines (17). We performed a systematic search from the following electronic databases: PubMed, EMBASE, and Cochrane Library, from their inception to January 20, 2021. The search terms were as follows: [1-chloro-4-(glucopyranos-1-yl)-2-(4-(tetrahydrofuran-3-yloxy)benzyl] benzene OR BI 10773 OR BI10773 OR BI-10773 OR Jardiance) AND (Cardiac Failure OR Heart Decompensation OR Decompensation, Heart OR Heart Failure, Right-Sided OR Heart Failure, Right Sided OR Right-Sided Heart Failure OR Right Sided Heart Failure OR Myocardial Failure OR Congestive Heart Failure OR Heart Failure, Congestive OR Heart Failure, Left-Sided OR Heart Failure, Left Sided OR Left-Sided Heart Failure OR Left Sided Heart Failure). No restrictions on language, publication date, or publication status were set in our review. We did not include conference abstracts. In addition, we reviewed the reference lists of eligible studies, previous review articles, and registered clinical trials.

### Study Selection and Eligibility Data

All searched articles were imported into EndNote software, and the title and abstract were screened by two reviewers (DP and PC). We entirely included clinical trials that met the following criteria for final analysis: (1) RCTs, (2) the target population was patients with HF, and (3) studies that included the comparison between empagliflozin and placebo.

The exclusion criteria were (1) observation, cohort, case control, case series, qualitative studies, uncontrolled trials, and laboratory studies and (2) duplicate studies with the same population (only the study with the largest participants was included in the meta-analysis if multiple studies included overlapping groups of patients).

If disagreements on study selection were identified, another author (MG) was consulted to solve them.

### Data Extraction

Two investigators (DP and PC) extracted the included trial data by using a predesigned form independently. The retrieved study characteristics were as follows: (1) first author's name and year of study; (2) study site; (3) intervention, dose of intervention, and comparison; (4) total sample size, and sample size of intervention and control group; (5) primary outcome; and (6) other outcomes.

### Outcomes

The primary outcome was determined as a composite of cardiovascular deaths and hospitalization for worsening heart failure. Other outcomes included hospitalization for worsening heart failure, changes in the NT-proBNP, 6WMT, or KCCQ score, and changes in body weight before and after the intervention. The endpoint definitions were those used in the individual trials.

### Risk of Bias Assessment

Assessment of the risk of bias for all of the included studies was performed independently by two review authors (DP and PC) through the Cochrane Risk of bias assessment tool. The assessment was performed across the following domains: selection bias (random sequence generation and allocation concealment), performance bias (blinding of patients and investigators), detection bias (blinding of outcome assessors), attrition bias (flawed outcome data), and reporting bias (selective reporting). According to the influence on material biases, the risk of bias was then adjudicated, with low, high, or unclear levels. To evaluate the certainty of the evidence for each outcome, we applied the Grading of Recommendations Assessment, Development and Evaluation (GRADE) Framework. All disagreements were resolved by the other two reviewers (MG and DS).

### Data Synthesis

We pooled the study results in the meta-analysis using Review Manager software (version 5.4.1; Nordic Cochrane Center, Cochrane Collaboration).

For dichotomous variables, risk ratios (RRs) with 95% confidence intervals (CIs) were calculated. Continuous variables were calculated and expressed in terms of mean differences (MD) and standard deviation (SD). For the articles that expressed continuous outcomes by median and interquartile range (IQR), the mean value and SD value were by the method of Luo et al. and Wan et al. (18, 19). For the articles that reported only the results before and after the intervention, the corresponding change from baseline was calculated according to the Cochrane Handbook for Systematic Reviews of Interventions. In the articles that reported mean and SD before intervention (M_baseline_, SD_baseline_), mean and SD after intervention (M_final_, SD_final_), and mean and SD of the change before and after intervention (M_change_, SD_change_), the correlation coefficient (Corr) was calculated as ${\text{SD}}_{\text{baseline}}^{2}$ + ${\text{SD}}_{\text{final}}^{2}$ – ${\text{SD}}_{\text{change}}^{2}$/2 * SD_baseline_ * SD_final_. For the articles that did not report the values of M_change_ and SD_change_, M_change_ and SD_change_ were calculated by the following equations: M_change_ = M_final_ – M_baseline_, SD_change_ = [${\text{SD}}_{\text{baseline}}^{2}$ + ${\text{SD}}_{\text{final}}^{2}$ – (2 * Corr * SD_final_ * SD_baseline_)]^1/2^. We assessed heterogeneity by using the X^2^ test and I^2^ statistic. Significant heterogeneity was defined as *I*^2^ > 50% with a *P* < 0.10. We used the random-effects model to estimate the pooled effect size from the included data if significant heterogeneity was observed across the trials, and the fixed-effects model was used otherwise. By conducting sensitivity and subgroup analysis, we probed sources of heterogeneity. Sensitivity analyses were conducted by one-by-one elimination. Publication bias was assessed using funnel plots if the included trial number was reasonable (10 or more). The quality of evidence for the main outcomes was assessed by the GRADE (20).

## Results

### Study Selection

**Figure 1:**
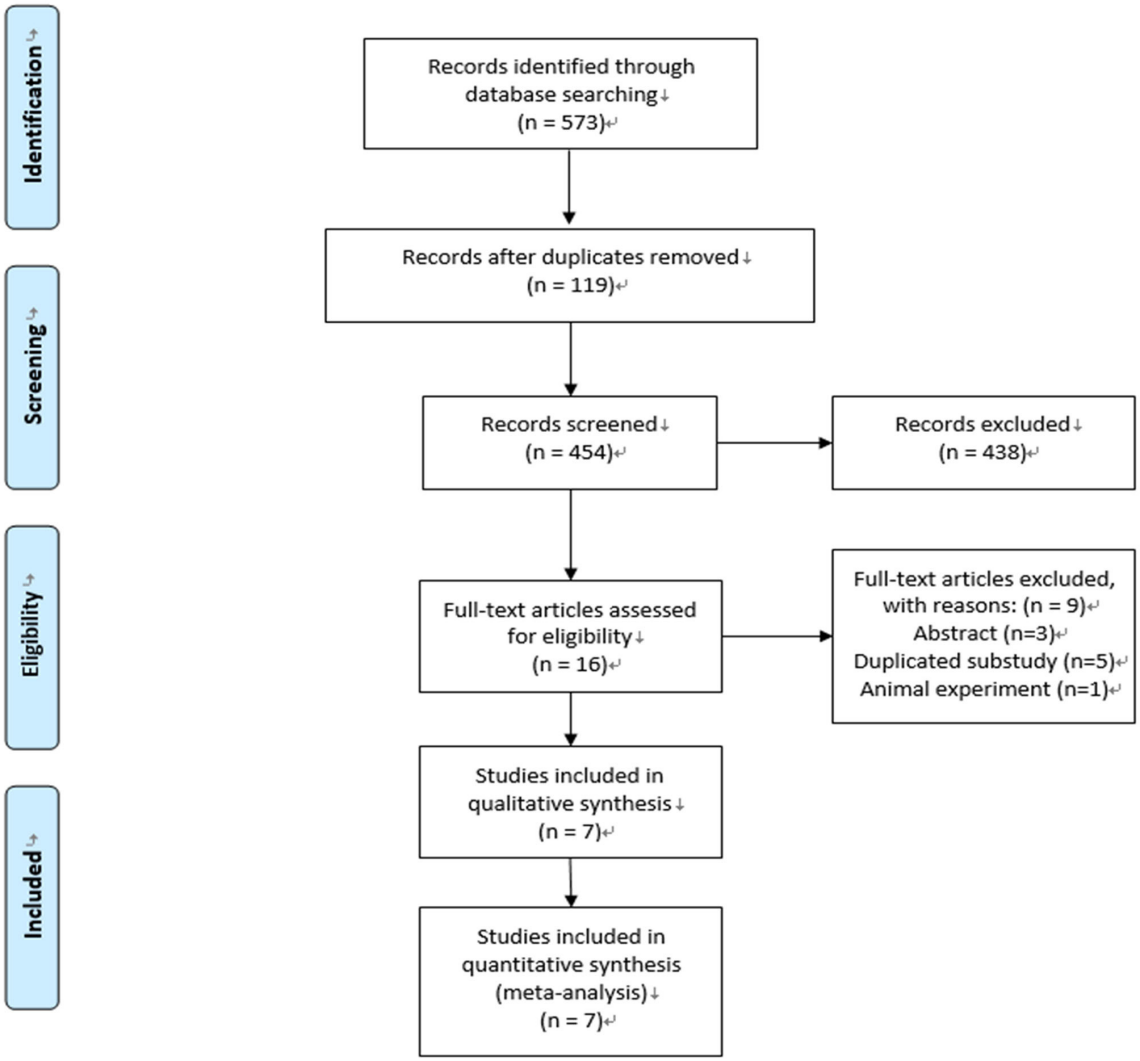
Flow chart of study selection and identification.

We conducted the last search on January 20, 2021, and the results of our literature search are shown in Figure 1. A total of 454 publications were identified. After the removal of duplications and screening, 16 studies were selected for eligibility by full-text screening, and seven trials were included in our systematic review and meta-analysis (11–15, 21, 22).

### Study Characteristics

Table 1 reports the characteristics of the included studies. Of the selected seven trials, 5,150 participants were included in total, 2,682 of them were in the empagliflozin group, and 2,468 of them were in the placebo group. Among them, six studies (11–15, 22) were two-arm RCTs. The last was a three-arm RCT (21) comparing two different doses (10 and 25 mg) of empagliflozin with placebo, and it was also the only substudy of a previous trial (9). In the selected trials, Packer's RCT (11) was the study with the largest sample size (*n* = 3,730). The age ranged from 59 to 70 years old, and the proportion of males was higher than that of females in all of the trials, ranging from 63% to 87%. Except for the three-arm RCT (21) and Mordi et al. (13), the dose of empagliflozin in the remaining trials was 10 mg once daily. The median follow-up time ranged from 6 weeks to 3.1 years.

**Table 1:** Characteristics of included studies.

**Article**	**Study site**	**Inclusion criteria**	**Sample size (empagliflozin/placebo)**	**Age**	**Male sex (%)**	**Body mass index**	**Intervention**	**Dose**	**Median time of follow-up**	**Primary outcome**	**Other outcomes**
Packer et al. (11)	Argentina, Australia, Belgium, Brazil, Canada, China, Czech Republic, France, Germany, Hungary, India, Italy, Japan, Mexico, Netherlands, Poland, Korea, Spain, United Kingdom, United States of America	Adults (≥18 ears of age) who had chronic heart failure (functional class II, III, or IV) with a left ventricular ejection fraction of 40% or less	3,730 (1,863/1,867)	Empagliflozin: 67.2 ± 10.8 placebo:66.5 ± 11.2	Empagliflozin: 74.5% placebo: 75.6%	Empagliflozin: 28.0 ± 5.5 placebo: 27.8 ± 5.3	Empagliflozin	10 mg, once daily	16 months	A composite of cardiovascular deaths or hospitalizations for worsening heart failure	Hospitalizations for heart failure, the rate of the decline in the estimated GFR, a composite renal outcome, total hospitalizations for any reason, and quality of life
Jensen et al. (12)	Denmark	Optimal HF therapy in accordance with European and national guidelines, LVEF ≤ 0.40, eGFR > 30 ml/min/1.73 m^2^, BMI <45 kg/m^2^, NYHA functional classes I–III, age > 18 years	190 (95/95)	Empagliflozin: 64 (57–73) placebo: 63 (55–72)	Empagliflozin: 83% placebo: 87%	Empagliflozin: 29 (23–29) placebo: 29 (23–30)	Empagliflozin	10 mg, once daily	12 weeks	NT-proBNP	Changes in hematocrit, systolic blood pressure, body weight, and estimated glomerular filtration rate (eGFR) from baseline to 12 weeks
Santos-Gallego et al. (15)	America	(1) Age >18 years; (2) Diagnosis of heart failure (NYHA II–III); (3) LVEF <50%; (4) Stable symptoms and medical therapy within the last 3 months	84 (42/42)	Empagliflozin: 64.2 ± 10.9 placebo: 59.9 ± 13.1	Empagliflozin: 63% placebo: 64%	Empagliflozin: 29.3 ± 6 placebo: 30 ± 6	empagliflozin	10 mg, once daily	6 months	LVEDV,LVESV	Changes in peak VO_2_, LV mass, LV ejection fraction (LVEF), LV sphericity index, oxygen uptake efficiency slope (OUES), VE/VCO_2_, distance in the 6MWT, and QoL (KCCQ-12)
Pellicori et al. (21)	Argentina, Australia, Austria, Belgium, Brazil, Canada, Colombia, Croatia, Czech Republic, Denmark, Estonia, France, Georgia, Greece, Hungary, Hong Kong, India, Indonesia, Israel, Italy, Japan, Korea, Malaysia, Mexico, Netherlands, New Zealand, Norway, Peru, Philippines, Poland, Portugal, Romania, Russia, Singapore, South Africa, Spain, Sri Lanka, Taiwan, Thailand Ukraine, United Kingdom, United States of America	T2D and established CV disease, narrow standardized Medical Dictionary for Regulatory Activities query (SMQ) “cardiac failure”	706 (462/244)	NA	NA	NA	Empagliflozin	10 or 25 mg, once daily	3.1 years	HF hospitalizations, CV death or HF hospitalizations, HF hospitalizations or all-cause mortality	Glycated hemoglobin, systolic blood pressure, and body weight
Lee et al. (22)	Scotland	18 years or older with HFrEF and type 2 diabetes [documented history of diabetes or previously undiagnosed diabetes with HbA1c ≥48 mmol/mol (≥6.5%)] or prediabetes (HbA1c 39–47 mmol/mol (5.7–6.4%) were potentially eligible. Participants had to be in the New York Heart Association (NYHA) functional classes II to IV and have a left ventricular ejection fraction (LVEF) ≤ 40%	105 (52/53)	Empagliflozin: 68.2 ± 11.7 placebo: 69.2 ± 10.6	Empagliflozin: 65.4% placebo: 81.1%	Empagliflozin: 30.9 ± 5.9 placebo: 30.4 ± 5.1	Empagliflozin	10 mg, once daily	36 weeks	LV end-diastolic volume (LVEDV) and LV end-systolic volume (LVESV)	Peak VO_2_, LV mass, LV ejection fraction (LVEF), LV sphericity index, oxygen uptake efficiency slope (OUES), VE/VCO_2_, distance in the 6MWT, and QoL (KCCQ-12)
Abraham et al. (14)	Australia, Canada, Germany, Greece, Italy, Norway, Poland, Portugal, Spain, Sweden, United States of America	Symptomatic (NYHA II–IV) HF diagnosed ≥3 months prior to screening with LVEF ≤ 40%, and 6-min walk test distance (6MWTD) of ≥100 m at baseline and ≤ 350 m at screening	312 (156/156)	Empagliflozin: 69.0 (62.5–77.0) placebo: 70 (62.5–77.0)	Empagliflozin: 77.6% placebo: 71.2%	Empagliflozin: 29.2 (25.7–32.9) placebo: 30.0 (26.4–33.6)	Empagliflozin	10 mg, once daily	12 weeks	6MWT	6MWTD, KCCQ-TSS responders (≥5 and ≥8 points) at week 12, change from baseline at week 12 in clinical congestion score, NT-proBNP, and intensification of diuretic use
Mordi et al. (13)	United Kingdom	18–80 years with previously diagnosed T2D, with a documented diagnosis of HF, in NYHA functional classes II–III, with prior echocardiographic evidence of heart failure with a left ventricular ejection fraction (LVEF) <50%	23 (12/11)	NA	NA	NA	Empagliflozin	25 mg, once daily	6 weeks	24-h urine volume	Change in 24-h urinary sodium (mmol/L and mmol/day), fractional excretion of sodium (FENa), assessment of electrolyte-free water clearance (ml), changes in renal biomarkers (serum creatinine, eGFR, and cystatin C, urine protein/creatinine and albumin/creatinine ratios), change in NT-proBNP

*BMI, body mass index; CV, cardiovascular; eGFR, estimated glomerular filtration rate; HF, heart failure; HFrEF, heart failure with reduced ejection fraction; NA, not available; KCCQ, The Kansas City Cardiomyopathy Questionnaire; KCCQ-TSS, The Kansas City Cardiomyopathy Questionnaire—Total symptom score; LVEF, left ventricular ejection fraction; LVEDV, left ventricular end-diastolic volume; LVESV, left ventricular end-systolic volume; NYHA, New York Heart Association; T2D, type 2 diabetes; VO_2_, oxygen consumption; VCO_2_, carbon dioxide production; VE, minute ventilation; 6MWT, 6-minute walk test*.

The outcomes included a composite of cardiovascular death or hospitalization for worsening heart failure [three RCTs (11, 12, 21)], hospitalization for worsening heart failure [three RCTs (11, 12, 21)], change in KCCQ score [three RCTs (11, 12, 14)], change in NT-proBNP from baseline [five RCTs (11–14, 22)], change in 6MWT from baseline [three RCTs (14, 15, 22)], and change in body weight from baseline [four RCTs (11–13, 15)].

### Assessment of Risk of Bias

All seven included studies reported the generation of random sequences, and all of them also provided generation methods. However, two studies (12, 14) did not report the method of allocation concealment, so their risk level remained unclear. One study (22) did not report the blinding of both treatment providers and participants, while the other studies adopted a double-blind design. Two studies (11, 12) reported blinding of the outcome assessment. All of the studies were evaluated as having a low risk of attrition bias with no loss to follow-up or low and balanced loss to follow-up. There was no selective bias in any of the seven studies. We also detected no other risk in the seven included studies.

### Results of Meta-Analysis

#### Primary Outcome

**Figure 2:**
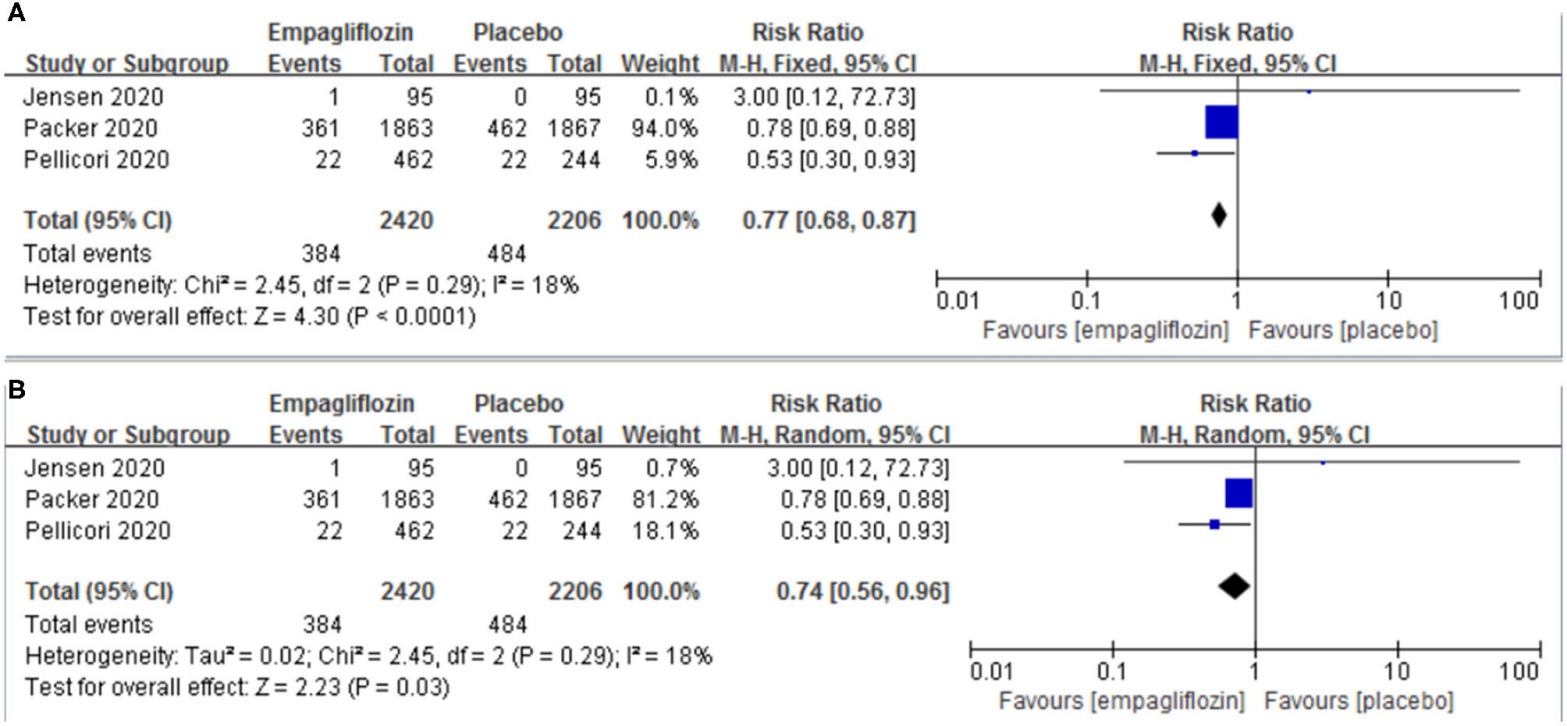
Effect of empagliflozin on a composite of cardiovascular deaths or hospitalizations for worsening heart failure [**(A)** by fixed-effects model; **(B)** by random-effects model].

Three studies (11, 12, 21) reported the result of a composite of cardiovascular death or hospitalization for worsening heart failure. Empagliflozin significantly reduced the risk of a composite of cardiovascular death or hospitalization for worsening heart failure compared with the placebo group [RR: 0.77 (95% CI 0.68–0.87]; *I*^2^ = 18%; *P* < 0.0001; Figure 2A]. We also conducted a meta-analysis by a random-effects model, and the result was consistent [RR: 0.74 (95% CI 0.56–0.96); *I*^2^ = 18%; *P* = 0.003; Figure 2B].

#### Other Outcomes

##### Hospitalization for Worsening Heart Failure

**Figure 3:**
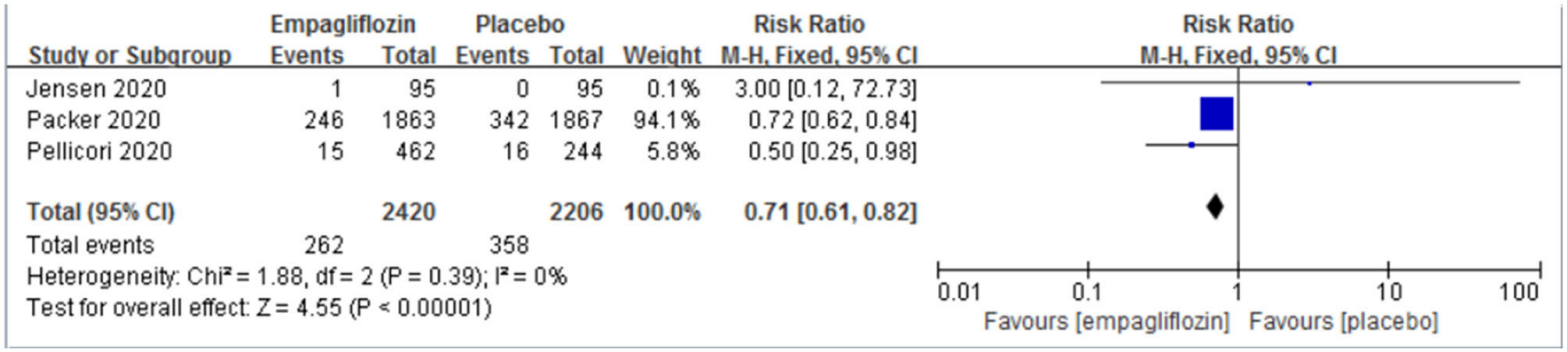
Effect of empagliflozin on hospitalization for worsening heart failure.

Three studies (11, 12, 21) reported the result of hospitalization for worsening heart failure, and we observed a significant difference between the two groups, which favored the empagliflozin group [RR: 0.71 (95% CI 0.61–0.82); I^2^ = 0%; P < 0.00001; Figure 3].

##### Six-Minute Walk Test

**Figure 4:**
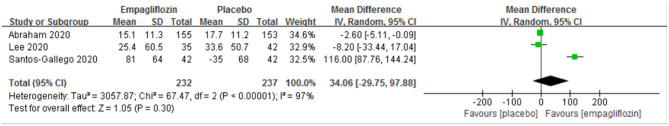
Effect of empagliflozin on the change in the 6-min walk test from baseline.

The outcome of the 6MWT was reported in three studies (14, 15, 22), and we did not observe a significant difference between the two groups with regard to the distance change in the 6MWT before and after intervention [mean difference (MD): 34.06 (95% CI, −29.75–97.88); *I*^2^ = 97%; *P* = 0.30; Figure 4].

##### NT-proBNP

**Figure 5:**
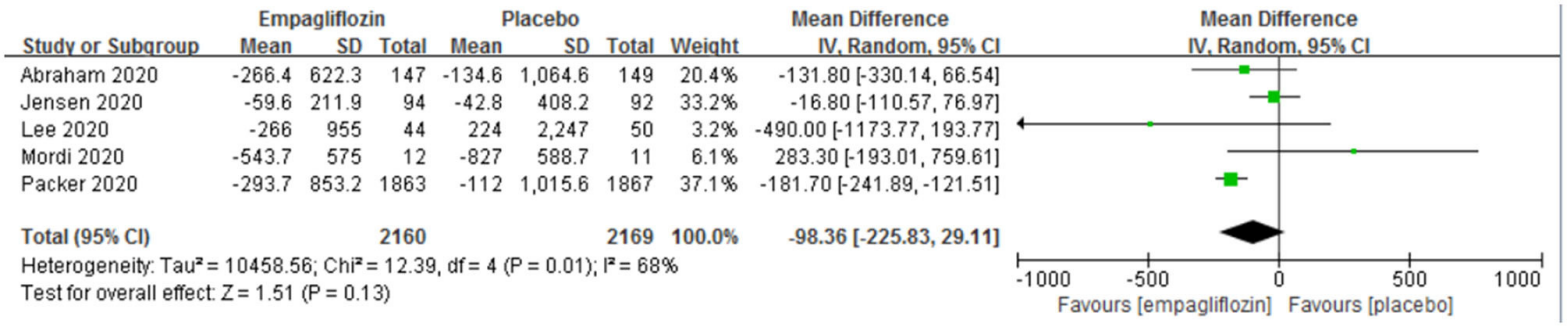
Effect of empagliflozin on the change in N-terminal pro-brain natriuretic peptide (NT-proBNP) from baseline.

The change from baseline of NT-proBNP was reported in five studies (11–14, 22), and no significant difference was observed between the two groups [MD: −98.36 (95% CI −225.83–29.11); *I*^2^ = 68%; *P* = 0.13; Figure 5].

##### KCCQ Score

**Figure 6:**
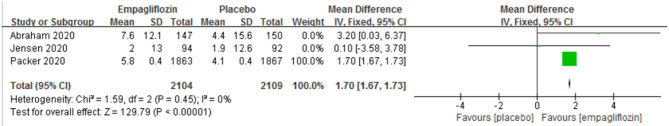
Effect of empagliflozin on the change in Kansas City Cardiomyopathy Questionnaire (KCCQ) score from baseline.

Three studies (11, 12, 14) reported the change in KCCQ score. A significant difference was observed between the empagliflozin group and the placebo group, which favored the empagliflozin group [MD: 1.70 (95% CI 1.67–1.73); *I*^2^ = 0%; *P* < 0.00001; Figure 6].

##### Body Weight

**Figure 7:**
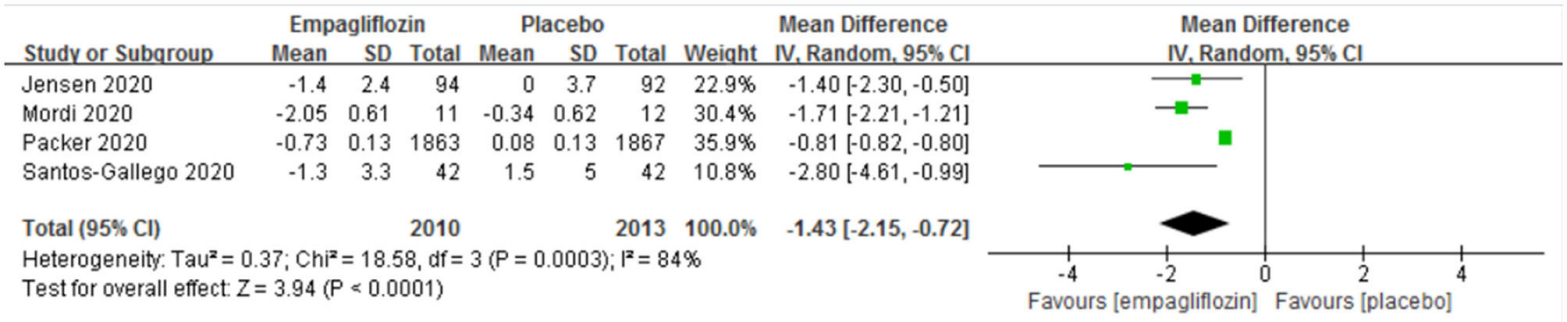
Effect of empagliflozin on the change in body weight from baseline.

Four studies (11–13, 15) reported the change in body weight before and after the intervention, and a significant difference was detected between the two groups [MD: −1.43 (95% CI, −2.15 to −0.72); *I*^2^ = 84%; *P* < 0.0001; Figure 7].

##### Subgroup Analysis

**Figure 8:**
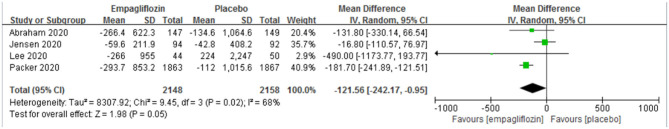
Effect of empagliflozin on the change in NT-proBNP from baseline in HFrEF patients.

**Figure 9:**
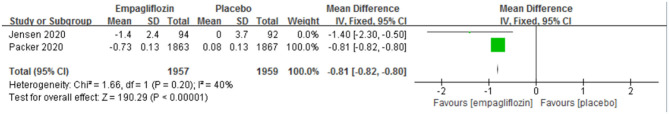
Effect of empagliflozin on the change in body weight from baseline in HFrEF patients.

Five RCTs (11, 12, 14, 21, 22) recruited patients with heart failure and reduced ejection fraction [defined as a left ventricular ejection fraction of <40% (31)]. In HFrEF patients, empagliflozin was associated with significantly better NT-proBNP reduction [MD: −121.56 (95% CI −242.17 to −0.95); *I*^2^ = 68%; *P* = 0.05; Figure 8]. In addition, empagliflozin showed a superior reduction in body weight in HFrEF patients [MD: −0.81 (95% CI −0.82 to −0.80); *I*^2^ = 40%; *P* < 0.00001; Figure 9].

##### Sensitivity Analysis

We performed sensitivity analysis to evaluate the influence of any individual study on the overall effect. For NT-proBNP, empagliflozin was significantly better than placebo when Jensen et al. (12) was removed [MD: −173.07 (95% CI −230.05 to −116.08); I^2^ = 35%; P < 0.00001]. For the 6MWT, the placebo group showed slightly better performance than the empagliflozin group when Santos-Gallego et al. (15) was removed [MD: −2.65 (95% CI −5.16 to −0.15); I^2^ = 0%; P = 0.04].

##### GRADE Assessment

Table 2 shows the GRADE assessment of the certainty of the effect of empagliflozin in patients with HF. Because the participants in one study were non-diabetic patients (15), whereas another study focused on patients with type 2 diabetes mellitus (13), the certainty level of body weight was downgraded to moderate. The certainty level of the 6MWT was downgraded to moderate for one article focused on non-diabetic patients (15), and another focused on patients with type 2 diabetes mellitus or prediabetes (22). The other outcomes, a composite of cardiovascular death or hospitalization for worsening heart failure, hospitalization for worsening heart failure, and changes in NT-proBNP and the KCCQ score, were evaluated as high certainty, with no evidence for downgrading.

**Table 2:** Grading of Recommendations Assessment, Development, and Evaluation (GRADE) assessment.

**Certainty assessment**							**No of patients**		**Effect**		**Certainty**
**No of studies**	**Study design**	**Risk of bias**	**Inconsistency**	**Indirectness**	**Imprecision**	**Other considerations**	**Empagliflozin**	**Placebo**	**Relative (95% CI)**	**Absolute (95% CI)**	
**A composite of cardiovascular death or hospitalization for worsening heart failure**											
3	Randomized trials	Not serious	Not serious	Not serious	Not serious	None	384/2,420 (15.9%)	484/2,206 (21.9%)	RR 0.77 (0.68 to 0.87)	51 fewer per 1,000 (from 71 fewer to 28 fewer)	⊕⊕⊕⊕ HIGH
**Hospitalization for worsening heart failure**											
3	Randomized trials	Not serious	Not serious	Not serious	Not serious	None	262/2,420 (10.8%)	358/2,206 (16.2%)	RR 0.71 (0.61 to 0.82)	47 fewer per 1,000 (from 64 fewer to 28 fewer)	⊕⊕⊕⊕ High
**6MWT**											
3	Randomized trials	Not serious	Not serious	Serious^a^	Not serious	None	232	237	–	MD 34.06 higher (29.75 lower to 97.88 higher)	⊕⊕⊕◯ Moderate
**NT-proBNP**											
5	Randomized trials	Not serious	Not serious	Not serious	Not serious	None	2,160	2,169	–	MD 98.36 lower (225.83 lower to 29.11 higher)	⊕⊕⊕⊕ High
**KCCQ**											
3	Randomized trials	Not serious	Not serious	Not serious	Not serious	None	2,104	2,109	–	MD 1.7 higher (1.67 higher to 1.73 higher)	⊕⊕⊕⊕ High
**Body weight**											
4	Randomized trials	Not serious	Not serious	Serious^b^	Not serious	None	2,010	2,013	–	MD 1.43 lower (2.15 lower to 0.72 lower)	⊕⊕⊕◯ Moderate

*CI, confidence interval; RR: risk ratio; 6MWT, 6-min walk test; KCCQ, kansas city cardiomyopathy questionnaire; MD, mean difference*.

a *One article included HF patients, all of whom were non-diabetic patients, and one article included HF patients with type 2 diabetes mellitus or prediabetes*.

b *One article included HF patients, all of whom were non-diabetic patients, and one article included HF patients with type 2 diabetes mellitus*.

## Discussion

### Summary of the Evidence

In this systematic review and meta-analysis, we included seven RCTs with 5,150 participants in total, and with regard to the primary outcome of a composite of cardiovascular death or hospitalization for worsening heart failure, the empagliflozin group showed significant superiority to the placebo group. In terms of hospitalization for worsening heart failure, the change in KCCQ score, and the change in body weight, significant differences were observed between the two groups and favored the empagliflozin group. For the results of the changes in 6MWT and NT-proBNP, we did not observe notable differences between the two groups except when studies that caused high heterogeneity were removed, when 6MWT favored the placebo, and NT-proBNP favored empagliflozin.

### Results in Relation to Other Studies

To our knowledge, this is the first systematic review and meta-analysis that concentrated on the effect of empagliflozini in patients with HF. Instead of assuming the parameter Corr to be 0.5, we calculated Corr, which made the results more convincing. We also included four new high-quality RCTs in our study and pooled two new outcomes (6MWT and body weight). Hence, our results provided more robust and comprehensive evidence for evaluating the effect of empagliflozin on HF. We observed significant improvements in heart failure hospitalizations and cardiovascular deaths, which is in line with a previous study (32) focused on the effect of SGLT2i in heart failure patients. However, regarding the two outcomes of a composite of cardiovascular death or hospitalization for worsening heart failure and hospitalization for worsening heart failure, our study showed greater RR reduction than the previous meta-analysis (32), which may indicate that empagliflozin is superior in patients with reduced ejection fraction. It is also worth noting that the results of our study and a previous trial with dapagliflozin intervention (33) demonstrated no significant difference in the change in NT-proBNP. However, some trials showed that SGLT2i, especially empagliflozin or dapagliflozin, were associated with a reduction in plasma NT-proBNP levels (11, 22, 30), which is consistent with the result of the HFrEF subgroup in our meta-analysis. Therefore, the results on NT-proBNP were controversial. However, with pooled results including new high-quality trials, we believe that our results are more convincing.

### Potential Mechanism

SGLT2i localize at the brush border of the early proximal tubule and function by reabsorbing nearly all of the filtered glucose (23, 24). Because the excretion of glucose was promoted, the blood glucose level was decreased. With lower blood glucose, lower heart failure mortality was observed in a cohort trial (25). However, the outcome cannot be simply explained by lower blood glucose, since other antidiabetic drugs with stronger effects did not show the same cardiovascular protective results (26). In addition to the aforementioned mechanism, SGLT2i prevents the reabsorption of glucose in the proximal tubule, further causing secondary osmotic effects and then leading to natriuretic and diuretic effects (23). This mechanism could account for the reduction in body weight, which was found in our study. Moreover, it has been proven that empagliflozin significantly improved left ventricular diastolic function and reduced mortality in mice, probably by reducing spontaneous diastolic sarcoplasmic reticulum calcium release. Leakage was thought to be the mechanism of diastolic dysfunction (27). In addition, empagliflozin also promoted cardiac function in non-diabetic rats, which may be associated with improved cardiac metabolism and cardiac ATP production (28).

Autophagy plays a crucial role in relieving cellular stress caused by different metabolites, such as glucose and lipids (29, 34), and clearing accumulated metabolites and dysfunctional organelles, thus, preventing cells from dysfunction or death. In heart failure patients, the autophagy capacity of cardiomyocytes is markedly impaired (35). Autophagic vacuoles, which reflect the extent of autophagy, were found to be associated with a better prognosis of heart failure (36). SGLT2i promotes urinary caloric loss, which mimics nutrient deprivation, in which autophagy is activated, leading to cellular survival, and homeostasis instead of growth (37). In non-diabetic mice, SGLT2i upregulated SIRT1 and AMPK, which promote autophagy, and downregulated the Akt-mTOR pathway, which has the opposite function (35, 37). Through the mechanisms mentioned above, autophagy was promoted, thereby protecting cardiomyocytes from dysfunction and death.

## Limitations

There are some limitations that should be noted. First, the doses and durations of follow-up in the included trials were not uniform. Consequently, we conducted sensitivity analysis to evaluate heterogeneity. The results of the 6MWT and NT-proBNP changed after sensitivity analysis, indicating that the two results should be interpreted with great caution. Second, the forms of reported results varied across trials, and the transformation to a consistent form may have introduced some inaccuracy. Nonetheless, we transformed the results according to relevant articles (18, 19), which minimized the error as much as possible. Third, we obtained trial-level data instead of individual-level data; thus, we could not conduct subgroup analysis based on type 2 diabetes mellitus, different drug therapies, New York Heart Association (NYHA) functional class, estimated glomerular filtration rate (eGFR), and so on.

## Conclusion

In conclusion, compared with placebo, empagliflozin significantly reduced a composite of cardiovascular deaths or hospitalizations for worsening heart failure but showed no statistically significant change in NT-proBNP. However, in HFrEF patients, empagliflozin showed a significant reduction in NT-proBNP. More high-quality, large-scale RCTs are needed to comprehensively evaluate the long-term effect of empagliflozin in patients with HF.

## Data Availability Statement

The original contributions presented in the study are included in the article/supplementary material, further inquiries can be directed to the corresponding author/s.

## Author Contributions

DP, LX, and MG designed the study, assessed the risk of bias, analyzed the data, and wrote the first and revised version of the manuscript. DP and PC screened and extracted the data. DP, DS, HJ, and MG modified the final manuscript. All authors read and approved the final manuscript, contributed to the conceptualization of the research questions, interpretation of the results, and article writing.

## Conflict of Interest

The authors declare that the research was conducted in the absence of any commercial or financial relationships that could be construed as a potential conflict of interest.
